# Lower-grade gliomas surgery guided by GRPR-targeting PET/NIR dual-modality image probe: a prospective and single-arm clinical trial

**DOI:** 10.7150/thno.91554

**Published:** 2024-01-01

**Authors:** Liangpeng Chen, Jingjing Zhang, Chongwei Chi, Wenqiang Che, Gehong Dong, Junmei Wang, Yanru Du, Rongxi Wang, Zhaohui Zhu, Jie Tian, Nan Ji, Xiaoyuan Chen, Deling Li

**Affiliations:** 1 Department of Neurosurgery, Beijing Tiantan Hospital, Capital Medical University, Beijing, China.; 2 Departments of Diagnostic Radiology, Surgery, Chemical and Biomolecular Engineering, and Biomedical Engineering, Yong Loo Lin School of Medicine and Faculty of Engineering, National University of Singapore, Singapore.; 3 Clinical Imaging Research Centre, Centre for Translational Medicine, Yong Loo Lin School of Medicine, National University of Singapore, Singapore.; 4 Nanomedicine Translational Research Program, NUS Center for Nanomedicine, Yong Loo Lin School of Medicine, National University of Singapore, Singapore.; 5 Key Laboratory of Molecular Imaging, Chinese Academy of Science, Beijing, China.; 6 Department of Neuropathology, Beijing Neurosurgical Institute, Capital Medical University, Beijing, China.; 7 Department of Pathology, Beijing Tiantan Hospital, Capital Medical University, Beijing, China.; 8 Department of Nuclear Medicine, Peking Union Medical College Hospital, Chinese Academy of Medical Sciences and Peking Union Medical College, China.; 9 China National Clinical Research Center for Neurological Diseases (NCRC-ND), Beijing, China.

**Keywords:** Lower-grade gliomas, Dual-modality imaging, Positron emission tomography, Near-infrared fluorescence, Intraoperative imaging, Gastrin-releasing peptide receptor

## Abstract

**Purpose:** Lower-grade gliomas (LGGs) are a group of infiltrative growing glial brain tumors characterized by intricate intratumoral heterogeneity and subtle visual appearance differences from non-tumor tissue, which can lead to errors in pathologic tissue sampling. Although 5-ALA fluorescence has been an essential method for visualizing gliomas during surgery, its effectiveness is limited in the case of LGGs due to low sensitivity. Therefore, we developed a novel PET/NIR dual-modality image probe targeting gastrin-releasing peptide receptor (GRPR) in glioma cells to enhance tumor visualization and improve the accuracy of sampling.

**Methods:** A prospective, non-randomized, single-center feasibility clinical trial (NCT 03407781) was conducted in the referral center from October 21, 2016, to August 17, 2018. Consecutive enrollment included patients suspected of having LGGs and considered suitable candidates for surgical removal. Group 1 comprised ten patients who underwent preoperative ^68^Ga-IRDye800CW-BBN PET/MRI assessment followed by intraoperative fluorescence-guided surgery. Group 2 included 42 patients who underwent IRDye800CW-BBN fluorescence-guided surgery. The primary endpoints were the predictive value of preoperative PET imaging for intraoperative fluorescence and the sensitivity and specificity of fluorescence-guided sampling.

**Results:** Thirty-nine patients were included in the in-depth analysis of endpoints, with 25 (64.1%) exhibiting visible fluorescence, while 14 (35.9%) did not. The preoperative positive PET uptake exhibited a greater accuracy in predicting intraoperative fluorescence compared to MRI enhancement (100% [10/10] *vs.* 87.2% [34/39]). A total of 125 samples were harvested during surgery. Compared with pathology, subjective fluorescence intensity showed a sensitivity of 88.6% and a specificity of 88.2% in identifying WHO grade III samples. For WHO grade II samples, the sensitivity and specificity of fluorescence were 54.7% and 88.2%, respectively.

**Conclusion:** This study has demonstrated the feasibility of the novel dual-modality imaging technique for integrated pre- and intraoperative targeted imaging via the same molecular receptor in surgeries for LGGs. The PET/NIR dual-modality probe exhibits promise for preoperative surgical planning in fluorescence-guided surgery and provides greater accuracy in guiding tumor sampling compared to 5-ALA in patients with LGGs.

## Introduction

Lower-grade gliomas (LGGs) constitute a group of slowly growing glial brain tumors characterized by diffuse infiltration and the potential for unpredictable malignant progression to higher grades 1, 2. The current standard for diagnosing and grading gliomas relies on pathological examination of resected specimens 3-5. However, the intricate intratumoral heterogeneity and subtle visual appearance differences from non-tumor tissue present significant challenges in precise pathologic sampling in patients with LGGs 5-7. Furthermore, a substantial proportion of LGGs lack notable enhancement on magnetic resonance imaging (MRI), providing no definite target for the localization of anaplastic foci, thereby reducing the reliability of tissue sampling 4, 6, 8. These limitations in tumor visualization may lead to suboptimal surgical planning and incorrect histopathological diagnosing and grading.

In the last decades, the utilization of 5-aminolevulinic acid (5-ALA) fluorescence for glioblastoma (GBM) intraoperative visualization has become a common practice 8-10. With promising outcomes observed in GBM, 5-ALA has been extended to investigations in LGGs over the last few years. Several studies have indicated that 5-ALA fluorescence might serve as a valuable marker for intraoperative detection of anaplastic foci in LGGs 11-13. However, the utilization of 5-ALA for LGGs is met with limited acceptance due to its notably low sensitivity, ranging from 52.9% to 77.6% in WHO grade III gliomas and from 0.0% to 25.0% in grade II gliomas 6, 12, 14. On the other hand, the intraoperative 5-ALA fluorescence in most LGGs remains unpredictable based on preoperative images. An earlier study suggested that 70.0% of the grade III gliomas without enhancement and 88.3% of tumors with enhancement on preoperative MRI can emit 5-ALA fluorescence intraoperatively 12. Differences in imaging targets lead to discrepancies between the MRI enhancement image and intraoperative fluorescence image, consequently limiting the MRI for selecting patients suitable for 5-ALA fluorescence-guided surgery.

An attractive strategy for achieving the visualization of LGGs is using an integrated preoperative positron emission tomography (PET) and intraoperative optical guidance dual-modality image probe that targets the same specific molecule in tumors 15. The gastrin-releasing peptide receptor (GRPR) is a promising specific target for gliomas, which can be effectively targeted by bombesin (BBN) 16, 17. Our team has successfully developed a novel PET and near-infrared (NIR) dual-modality image probe by conjugating BBN with the positron-emitting nuclides ^68^Ga and the NIR fluorescent dye IRDye800CW. This innovative probe has enhanced GBM visualization by integrating preoperative PET planning with real-time guidance through intraoperative NIR fluorescence. These advancements have played a crucial role in facilitating the attainment of maximum safe resection, as evidenced by positive outcomes in a clinical trial (NCT 02910804) 18, 19.

In this study, our objective was to investigate the feasibility of the GRPR-targeting PET/NIR dual-modality image probe in LGGs management. We aimed to assess the predictive value of preoperative ^68^Ga-IRDye800CW-BBN PET assessment for intraoperative fluorescence and to analyze the accuracy of fluorescence-guided sampling.

## Methods

### Patients

For this non-randomized, open-label, single-arm, single-center feasibility and safety study, eligible participants were adults suspected to have LGGs and considered suitable for surgical removal. The protocol specified that these patients should not exhibit typical imaging features of GBM on MRI, as assessed by the research team (neurosurgeons, neuroradiologists, and nuclear medicine physicians). Exclusion criteria included tumors originating from the brainstem, multifocal tumors, any contraindication of MRI or PET scanning, the Karnofsky performance scale (KPS) score of less than 60, renal or hepatic insufficiency, known allergy to contrast agents, the history of previous anaphylactic shocks, as well as lactating or pregnant women.

All patients provided written informed consent, and the study received approval from the committees of Beijing Tiantan Hospital and Peking Union Medical College Hospital. This study was registered at ClinicalTrials.com (NCT03407781).

### MRI assessment

All patients underwent an MRI scan (3T, Siemens) within two weeks before surgery, including a T1-weighted sequence and Gd-diethylenetriaminepentaacetic acid (Gd-DTPA, 20 mL) enhancement, T2-weighted sequence, and transverse fluid attenuation inversion recovery (FLAIR) sequence. The postoperative MRI was performed 72 hours after surgery.

The enhancement on MRI was classified into three categories: no enhancement, weak and patchy enhancement, and strong enhancement 20. The location of the tumor was classified as involving non-eloquent, near-eloquent, and eloquent, concerning their relationship with eloquent brain areas (e.g., region for motor control, sense, speech, cognition, or visual function) 9, 21. Tumor volume was calculated through the segmentation of individual scans, and the volumes were summed to obtain the total volume in FLAIR images. The extent of resection (EOR) was calculated using the formula: (preoperative tumor volume - postoperative tumor volume) / preoperative tumor volume 22.

### PET/MRI assessment

The synthesis of ^68^Ga-IRDye800CW-BBN followed previously reported procedures 17, 18, 23. Patients underwent PET/MRI and MRI scans no more than 7 days apart. A single dose of ^68^Ga-IRDye800CW-BBN (1.85 MBq per kilogram of body weight) was injected intravenously, and after 30 minutes, a PET/MRI scan covering the entire head of the patient was conducted. Two experienced nuclear medicine physicians qualitatively analyzed PET uptake of tumors, categorizing them as positive or negative, with each physician blinded to the result of the other. Quantitative lesion-specific uptake analysis was characterized by maximal and mean standardized uptake values (SUV_max_ and SUV_mean_, respectively) using a spherical volume of interest. The tumor-background uptake ratio (TBR) was defined as tumor SUV_mean_ / background SUV_mean_ 23.

### Fluorescence-guided Surgery

All patients received an intravenous infusion of 1.0 mg IRDye800CW-BBN (in 20 mL sterile water) 16 hours before the surgery 18. The intraoperative fluorescence camera system was a customized NIR fluorescence images system (DPM-I, Beijing Digital Precision Medicine Co., Ltd.), which was designed to capture the emission fluorescence signal at 795 nm with maximum efficiency based on the properties of IRDye800CW-BBN 18, 19, 24.

After tumor exposure, the DPM-I camera was positioned above the operation field to observe the fluorescence distribution in the tumor area. The fluorescence was visually categorized into three groups: no visible fluorescence, weak and patchy fluorescence, and strong fluorescence. This categorization was independently assessed by three neurosurgeons. During resection, the white light microscope (M205FA, Leica, Germany) was switched to the DMP-I for fluorescence visualization. Tissue biopsies were harvested from the tumor core, cavity, and non-eloquent area if neurosurgeons judged the procedure safe for the patient. Tumors were resected until all fluorescent lesions were removed, as determined by the surgeon. The fluorescence of extracted tissue was reconfirmed by DPM-I in a dark environment before securing samples in preprepared vials for pathology. Other intraoperative technical adjuncts (e.g., sonography or neuronavigation) were only permitted to plan the surgical approach or locate the initial tumor site.

The fluorescence intensity was then quantified offline using Fuji-Image J (Version 1.47, National Institution of Health, Bethesda, MD). For *ex vivo* sample fluorescence images, the signal-to-background ratio (SBR) of the samples was determined by dividing the mean signal intensity of the biopsied area by that of the background region 25.

### Neuropathology

The pathology criteria were graded according to the 2021 World Health Organization (WHO) classification of tumors of the central nerve system 26, 27. Immunohistochemistry or gene sequencing of Ki-67/MIB-1 proliferation index, IDH (R132H) mutation status, 1p19q co-deletion status, MGMT (O^6^-methylguanine DNA methyltransferase) promoter methylation status, and GRPR expression were performed as previously described 18, 19, 28, 29.

The pathology of the biopsy sample was independently determined by two neuropathologists who were blind to whether samples were fluorescent positive during surgery. In case of any discrepancy, a consensus was reached by involving another senior pathologist. The histopathology of biopsy samples was classified into the following groups: normal-appearing brain tissue, glioma-infiltrated tissue, frank tumor 30.

### Adjuvant therapy and follow-up

Decisions regarding adjuvant therapies were determined through evaluations conducted within our multidisciplinary tumor board. In cases of progression or malignant degeneration, patients received treatment according to established guidelines 31, 32.

Patients were monitored for progression-free survival (PFS) and overall survival (OS). Progression was defined according to RANO criteria by an independent neuroradiologist and was based on imaging with or without histological corroboration. Patients who died from any cause before documented progression were counted as an event for this endpoint 9.

### Safety assessment

Blood tests, including blood routine examination, complete chemistries, and metabolic panel, were conducted before and after the use of probes. Before each probe injection, an electrocardiogram and vital signs (blood pressure, heart rate, respiratory rate, and blood oxygenation) were recorded for patients. Any discomforts, such as nausea, vomiting, shortness of breath, etc., were documented. Temperature was recorded twice a day to rule out the pyrogenic effect. If the temperature exceeded 38.5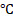, the suspected cause of fever was assessed for differential diagnoses, including postoperative central nervous system infection, pneumonia, urinary tract infection, etc.

### Primary and secondary endpoints

All enrolled patients underwent analysis for tracer-related adverse effects. Subsequently, for the in-depth analysis of additional endpoints, exclusive consideration was given to patients with histologically confirmed LGGs.

The primary endpoints of this study comprised the assessment of the predictive value of preoperative PET imaging for intraoperative fluorescence, as well as the evaluation of the sensitivity and specificity of fluorescence-guided sampling. Secondary endpoints included the identification of optimal SBR cutoff value for semi-quantification fluorescence intensity* ex vivo* to distinguish tumor and non-tumor tissues, the assessment of EOR, and the evaluation of PFS and OS, in addition to safety considerations.

### Statistical analysis

Normally distributed continuous variables were presented as mean 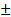 SD and compared using the Student's *t*-test; non-normally distributed continuous variables were expressed as median with interquartile ranges (IQRs) and compared using the Wann-Whitney *U* test. The Kruskal-Wallis test was used for multiple comparisons. Categorical variables were tested for baseline comparability with the chi-square or Fisher's exact test. The diagnostic performance of semi-quantification fluorescence intensity of samples to distinguish the tumor and non-tumor tissues was assessed via receiver-operating characteristic (ROC) analysis with the total area under the curve (AUC) calculated. Decision thresholds of SBR were determined based on the Youden index. Pooled diagnostic positive predictive value (PPV) and negative predictive value (NPV) were calculated for each subgroup of LGGs. Time-to-event measures, such as FPS and OS, were analyzed using the Kaplan-Meier methods, and comparisons were made using Log-rank tests. All analyses were conducted using SPSS (SPSS version 26.0; SPSS Inc, IL) with statistical significance levels set at *P* < 0.05.

## Results

**Figure 1:**
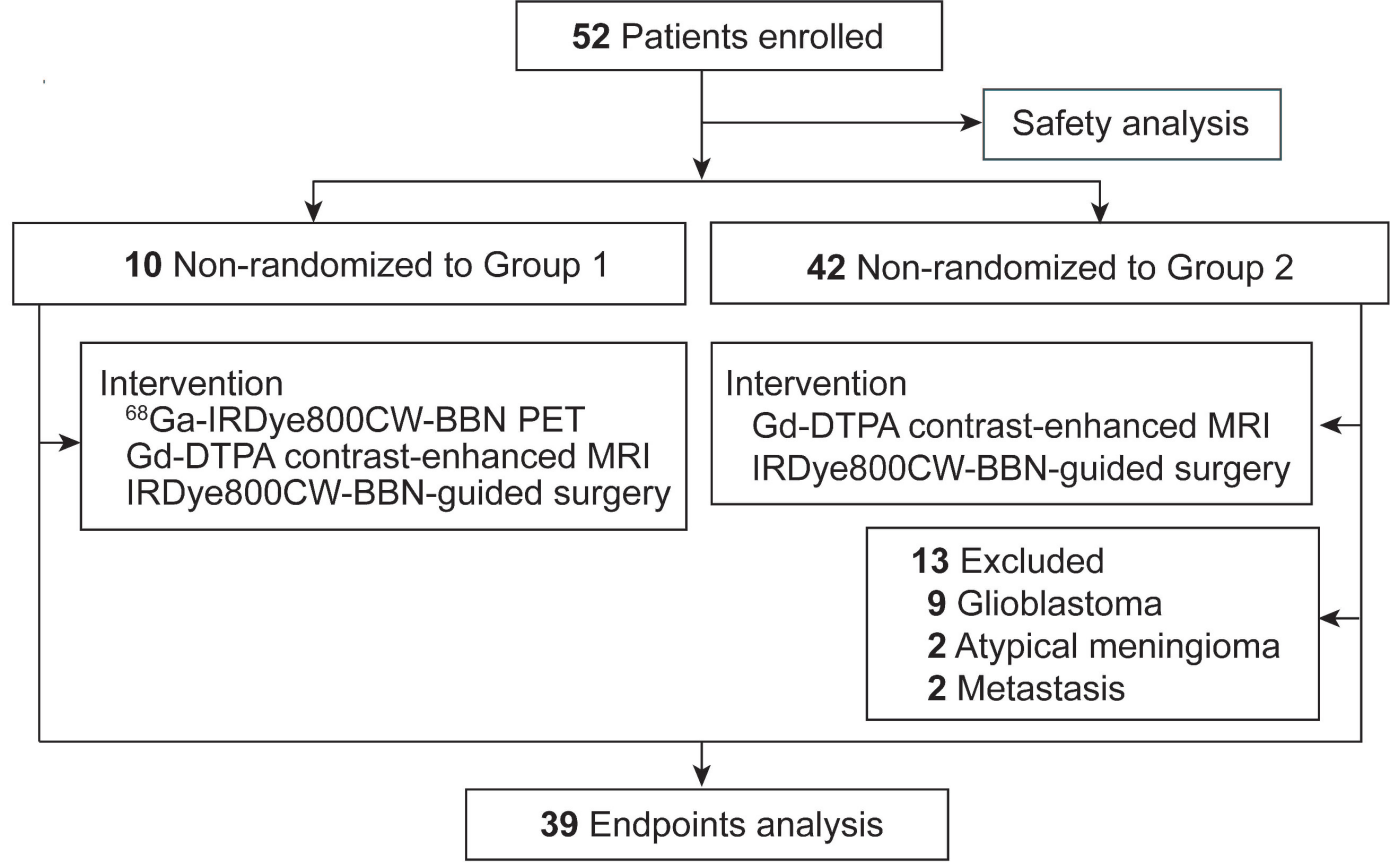
Study flowchart.

From Oct 21, 2016, to Aug 17, 2018, a total of 52 patients were prospectively enrolled in this trial (**Figure 1**). Group 1 comprised ten patients who underwent preoperative ^68^Ga-IRDye800CW-BBN PET assessment followed by intraoperative fluorescence-guided surgery. Group 2 included 42 patients who underwent IRDye800CW-BBN fluorescence-guided surgery. All enrolled patients were included in the tracer-related adverse effects analysis. Thirteen patients were excluded from the in-depth analysis of additional endpoints due to histologic diagnoses different from LGGs. Specifically, nine patients had GBM, two had atypical meningiomas, and two had metastases.

### Baseline clinical characteristics

Thirty-nine patients were included in the analysis of endpoints, with 25 patients (64.1%) in the fluorescence**-**positive group and 14 patients (35.9%) in the fluorescence-negative group. Among the fluorescence-positive patients, 23 exhibited patchy and weak fluorescence, while 2 displayed strong fluorescence. Baseline clinical characteristics revealed significant differences in age (39.3 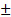 10.5 *vs.* 45.8 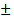
10.8, *P* = 0.035) and tumor volume (50.6 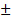 26.3 vs. 87.2 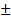 51.8, *P* = 0.019) between the fluorescent-negative and positive groups (**Table 1**). Notably, fluorescence was significantly associated with pathology WHO grade and Ki-67/MIB-1 index. Intraoperative fluorescence was more frequently observed in WHO grade III gliomas (89.5%, 17/19) than grade II gliomas (40.0%, 8/20; *P* = 0.002). The Ki-67/MIB-1 index in the fluorescent group was higher than the nonfluorescent group (21.0 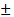
12.5* vs.* 8.3 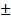 5.7; *P* = 0.001). Additionally, intraoperative fluorescence showed no correlation with IDH mutations, 1p19q co-deletion, or MGMT promoter methylation.

### Predictive value of preoperative PET imaging for intraoperative fluorescence

**Figure 2:**
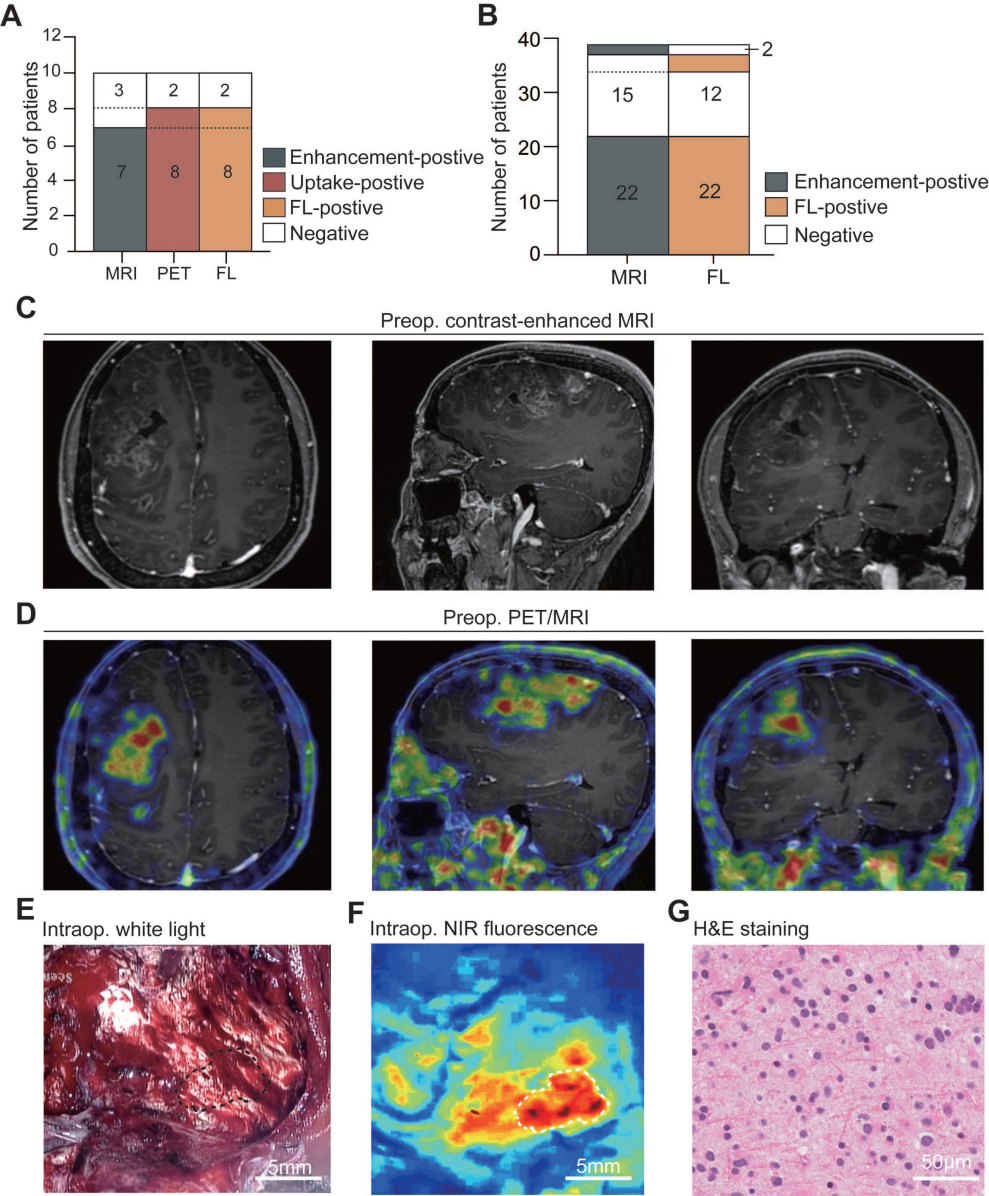
**Relationship between preoperative imaging and intraoperative fluorescence.** A. The relationship between enhancement on MRI, PET uptake, and intraoperative fluorescence (FL) in Group 1 (*n* = 10). B. The relationship between enhancement on MRI and intraoperative fluorescence in all patients (*n* = 39). C-G. Representative example of ^68^Ga-IRDye800-BBN PET/NIR dual-modality probe in LGGs management. The preoperative MRI showed a right frontal lobe glioma with weak and patchy enhancement (C). The preoperative PET showed positive uptake with a clear margin larger than that in contrast-enhanced MRI (D). During fluorescence-guided surgery, following partial tumor resection, the remaining tumor could not be differentiated under white light microscopy (indicated with black dashed line; E) but exhibited clear fluorescence when observed with the NIR imaging system (indicated with white dashed line; F). Subsequently, the fluorescent biopsy lesion was confirmed to be an astrocytoma (IDH-mutant, CNS 2; G).

In Group 1, eight out of 10 patients exhibited positive findings on preoperative PET and intraoperative fluorescence imaging, confirming diagnoses of oligodendrogliomas (IDH-mutant, and 1p19q-codeleted, CNS 3; *n* = 4), astrocytoma (IDH-mutant, CNS 3; *n* = 2), oligodendrogliomas (IDH-mutant, and 1p19q-codeleted, CNS 2; *n* = 1) and astrocytoma, (IDH-mutant, CNS 2; *n* = 1). The values of SUV_max_ and SUV_mean_ were 1.13 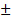 0.55 and 0.71 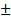 0.35, respectively. The mean TBR were 15.94 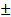 9.83. The remaining two patients exhibited negative uptake on PET and negative fluorescence during surgery, and their pathology confirmed astrocytoma (IDH-mutant, CNS 2). The accuracy of preoperative PET imaging in predicting intraoperative fluorescence was 100.0% (10/10; **Figure 2A**).

In Group 1 and Group 2, twenty-four out of 39 patients (66.7%) exhibited enhancement on MRI, including 22 patients displaying weak and patchy enhancement and two showing strong enhancement. In contrast, fifteen patients (33.3%) demonstrated no enhancement. The accuracy of preoperative enhancement on MRI in predicting intraoperative IRDye800CW-BBN fluorescence was 87.2% (34/39;** Figure 2B**). There was a mismatch between enhancement on MRI and intraoperative fluorescence in 5 patients. Specifically, two patients (one with WHO grade III gliomas and one with grade II) showed weak and patchy enhancement but no fluorescence. In contrast, three patients (two with WHO grade III and one with grade II glioma) displayed no enhancement on MRI but exhibited fluorescence. Notably, only one of the three patients underwent preoperative PET assessment and presented avid uptake on ^68^Ga-IRDye800CW-BBN PET assessment with a confirmed pathologic diagnosis of WHO grade III gliomas; unfortunately, the remaining two patients did not undergo PET assessment.

### Sensitivity and specificity of fluorescence-guided sampling

**Figure 3:**
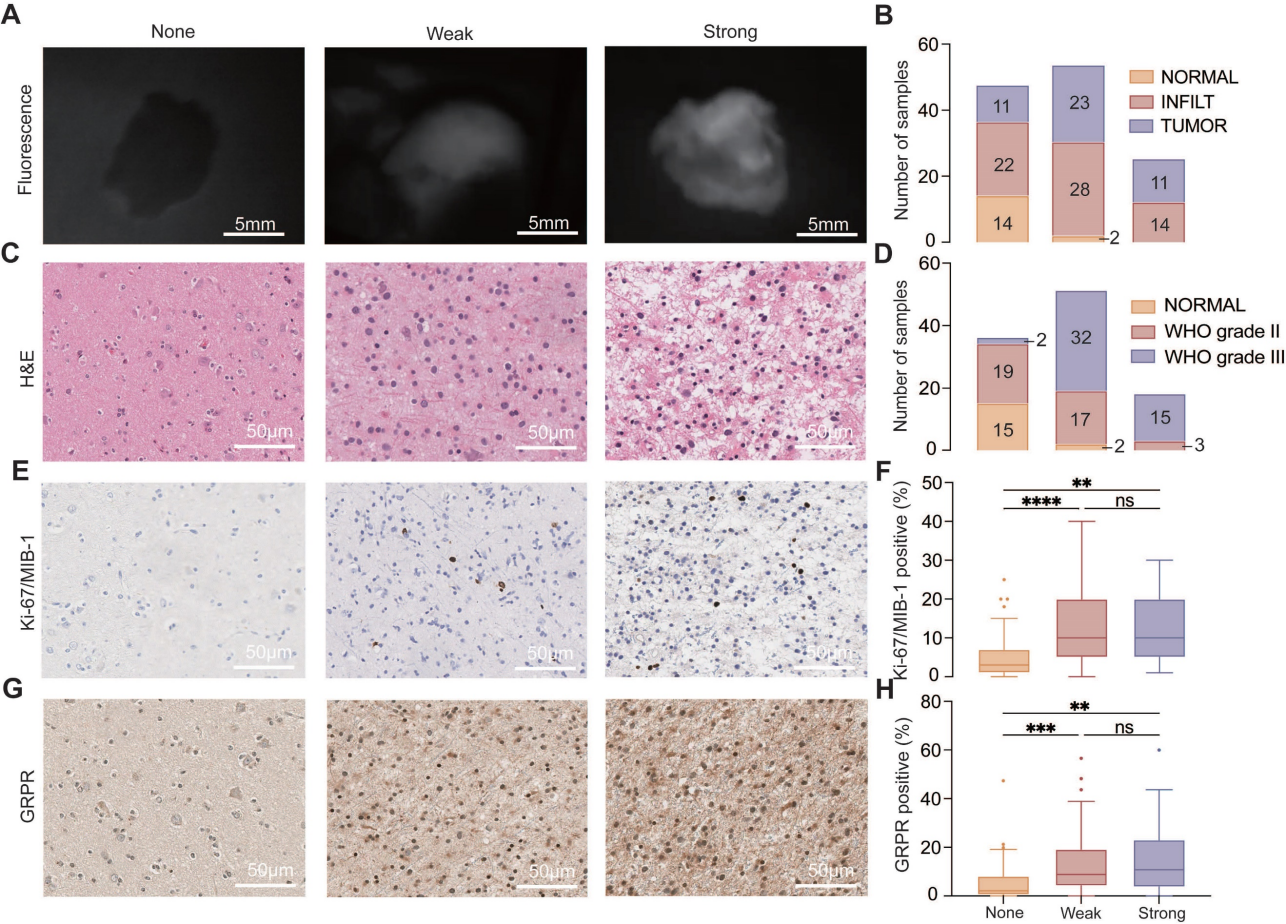
**Visual fluorescence image and pathology of biopsy samples.** A. Representative image of none, weak, and strong fluorescence samples under illumination with the NIR fluorescence imaging system. B. Biopsy specimens were classified as follows: normal-appearing brain tissue (NORMAL), glioma-infiltrated tissue (INFILT), and frank tumor (TUMOR). The distribution of pathology classification based on fluorescence intensity is graphed for all 125 samples. C. Representative images of H&E staining for none, weak, and strong fluorescence samples. D. Biopsy specimens were classified as 2021 WHO classification of tumors of the central nerve system. The distribution of pathology grade aligned with fluorescence intensity is graphed for all 125 samples. E. Representative images of Ki-67/MIB-1 staining of none, weak, and strong fluorescence samples. F. Quantitative analysis of Ki-67/MIB-1 (None, 3.0 [IQRs, 1.0 - 7.0]; weak, 10.0 [IQRs, 5.0 - 20.0]; strong, 10.0 [IQRs, 5.0 - 20.0]). G. Representative images of GRPR expression for none, weak, and strong fluorescence samples. H. Quantitative analysis of GRPR expression (None, 2.09 [IQRs, 0.46 - 8.12]; weak, 8.84 [IQRs, 4.17 - 19.17]; strong, 10.75 [IQRs, 7.72 - 23,07]). ***,* P* < .01, ****, *P* < .001; ns, *P* > .05 not significant.

A total of 125 samples were harvested during surgery. Visible fluorescence was observed in 78 samples (62.4%), comprising 53 samples (42.4%) with weak and patchy fluorescence, 25 samples (20.0%) exhibiting strong fluorescence, while 47 samples (36.6%) showed no fluorescence (**Figure 3A**). These samples were categorized into three groups based on histopathology: frank tumors (*n* = 44), glioma-infiltrated brain tissues (*n* = 65), and normal-appearing brain tissues (*n* = 16; **Figure 3B-C**). Tumor-containing biopsies included both frank tumors and glioma infiltration, classified according to WHO grade criteria, with 38.5% (42/109) classified as grade III gliomas and 61.5% (67/109) as grade II (**Figure 3D**).

With these parameters, subjective fluorescence intensity demonstrated a sensitivity of 88.6% and a specificity of 88.2% in identifying grade III tumor samples, yielding a positive predictive value (PPV) for pathology of 95.1% and a negative predictive value (NPV) of 75.0%. For grade II samples, the sensitivity and specificity of fluorescence were 54.7% and 88.2%, respectively, with a PPV of 94.6% and an NPV of 34.1%. Importantly, the Ki-67/MIB-1 index and GRPR expression were significantly higher in the weak and strong fluorescence groups compared to the non-fluorescence group (all *P* < 0.001; **Figure 3E-H**), but no statistically significant differences were observed between weak and strong fluorescence group.

### Semi-quantification fluorescence intensity analysis of samples

**Figure 4:**
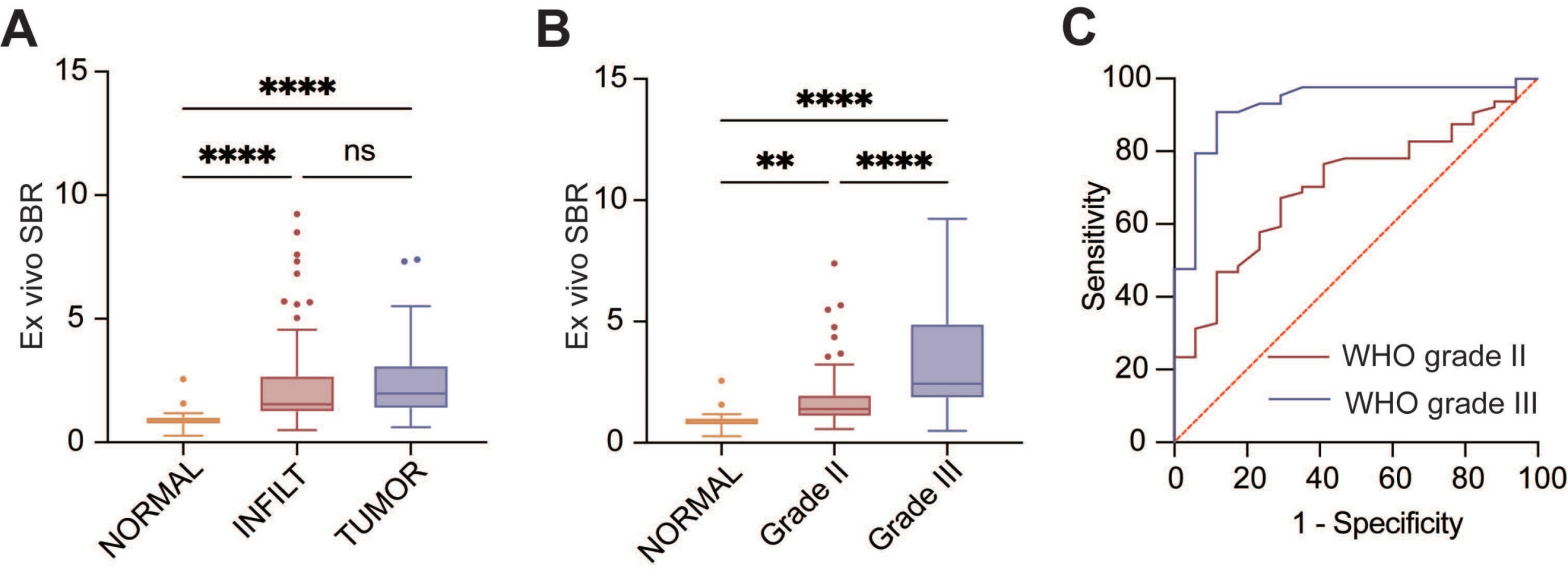
** Semi-quantitative analysis of fluorescence intensity of samples (*n* = 125).** A. Semi-quantitative fluorescence analysis classified samples into normal-appearing brain tissue (NORMAL, 0.91 [IQRs, 0.71 - 1.03]), glioma-infiltrated tissue (INFILT, 1.54 [IQRs, 1.21 - 2.70]), and frank tumor (TUMOR, 1.97 [IQRs, 1.35 - 3.12]). B. Semi-quantitative fluorescence analysis based on 2021 WHO classification of tumors of central nerve system (WHO grade II, 1.39 [IQRs, 1.07 - 1.99]; WHO grade III, 2.45 [IQRs, 1.83 - 4.92]). C. ROC analysis for WHO grade II and III glioma samples. WHO grade III gliomas, AUC 0.93 (95% CI, 0.85 - 1.00); WHO grade II gliomas, AUC 0.69 (95%CI, 0.56 - 0.81). ***,* P* < .01, ****, *P* < .001; ns, *P* > .05 not significant.

The semi-quantification fluorescence intensity analysis of samples was further assessed based on histopathological results. The median SBR for frank tumors, glioma-infiltration, and normal-appearing brain tissue were 1.97 (IQRs, 1.35 - 3.12), 1.54 (IQRs, 1.21 - 2.70), 0.91 (IQRs, 0.71 - 1.03), respectively. Non-tumor tissue exhibited lower fluorescence intensity compared to glioma-infiltrated brain tissue (*P* < 0.001) and frank tumor (*P* < 0.001; **Figure 4A**). Furthermore, the median SBR of grade II and III gliomas were 1.39 (IQRs, 1.07 - 1.99) and 2.45 (IQRs, 1.83 - 4.92), respectively. The SBR of grade III gliomas was significantly higher than grade II gliomas (*P* < 0.001) and normal tissue (*P* < 0.001). A statistically significant difference in SBR was also observed between grade II gliomas and normal tissue (*P* = 0.007; **Figure 4B**).

In the ROC analysis of the SBR to distinguish WHO grade III gliomas from non-tumor tissues, an optimal threshold value of 1.35 was identified (AUC 0.93 [95% CI, 0.85 - 1.00]; **Figure 4C**). The sensitivity and specificity of fluorescence were 90.9% and 88.2%, respectively. The PPV and NPV were 95.2% and 78.9%, respectively. For differentiating WHO grade II gliomas and non-tumor tissues in the ROC analysis, the best cutoff value of SBR was determined to be 1.00 (AUC 0.69 [95%CI, 0.56 - 0.81]). The results showed a sensitivity of 78.1%, specificity of 58.5%, PPV of 87.7%, and NPV of 41.7%.

### EOR and survival analysis

The resection percentage of FLAIR-abnormal volume in the fluorescence-positive group did not differ significantly from the negative group (74.8% [IQRs, 64.3% - 84.4] *vs.* 80.0% [IQRs, 72.1% - 84.4%], *P* = 0.593). Lesions with visual fluorescence were completely resected in all patients except for one case involving an anaplastic oligoastrocytoma spanning both sides of the frontal lobes and corpus callosum and another case of anaplastic astrocytoma located near the basal ganglia.

**Figure 5:**
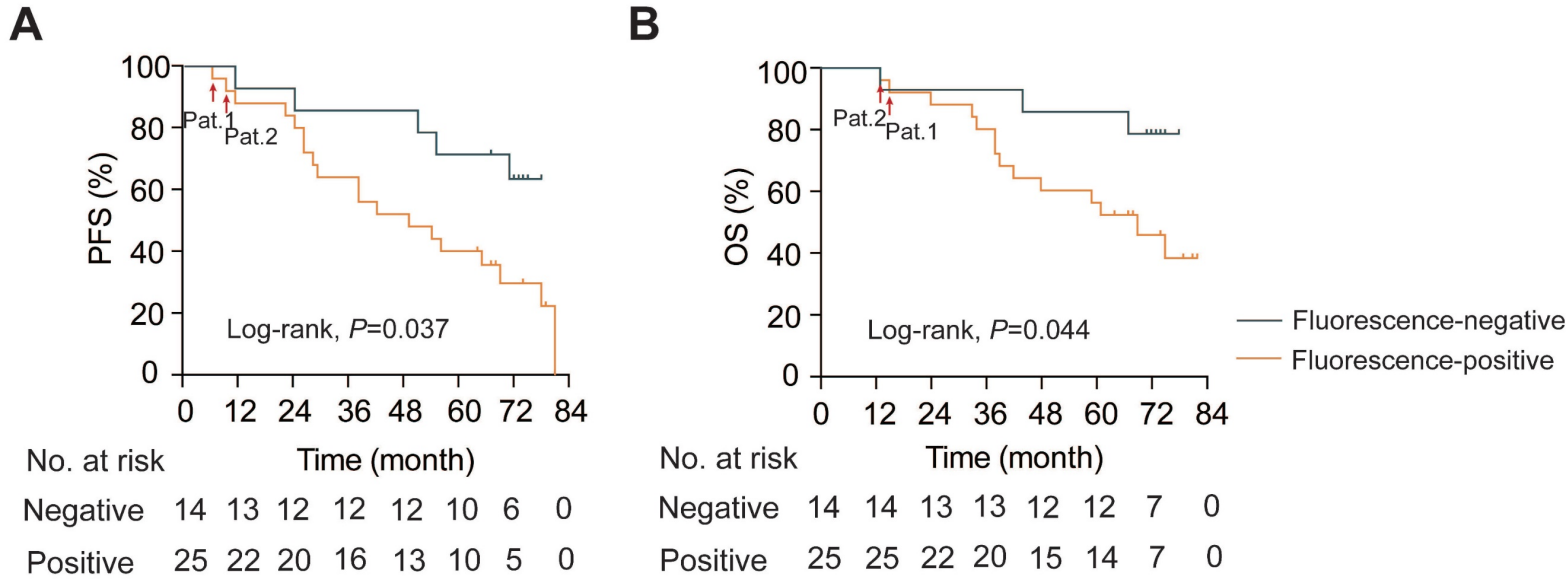
** Kaplan-Meier survival analysis.** A. Progression-free survival analysis (PFS; Log-rank, *P* = 0.037). B. Overall survival analysis (OS; Log-rank, *P* = 0.044). Pat.1 and Pat.2: patients with residual fluorescent lesions after resection.

The median follow-up was 67.0 months (IQRs, 38.0 - 74.0). In patients with intraoperative fluorescence, the PFS was 49.0 months (95% CI 22.9 - 75.1), while the median PFS in the fluorescence-negative group did not reach (log-rank, *P* = 0.037; **Figure 5A**). Similarly, the median OS in the fluorescence-negative group did not reach, but the positive group exhibited a shorter median OS (69.0 months, 95% CI 50.5 - 87.5; log-rank, *P* = 0.044; **Figure 5B**). Notably, the two patients with residual fluorescent foci after tumor resection experienced much shorter PFS and OS compared to patients without residual visual fluorescence lesions (Pat.1 and Pat.2. in **Figure. 5**).

### Safety and adverse effects

No anaphylactic reactions were reported about ^68^Ga-IRDye800CW-BBN. One patient reported transient nausea and increased heart rate, while the other nine patients did not experience any discomfort. Additional safety aspects, including hematological, liver, and renal toxicities of the probe, are presented in **Figure S1**.

Throughout the entire study period involving all 52 participants, no adverse events related to IRDye800CW-BBN were observed. The most prevalent systemic symptoms were mild nausea (65.4%, 34/52) and sinus tachycardia (42.3%, 22/52), followed by abdominal pain (5.8%, 3/52) and chest tightness (3.9%, 2/52). All these discomforts were relieved within 15 minutes. Sixteen patients developed a fever within 3 to 7 days after surgery, and clinical manifestations and cerebrospinal fluid examination confirmed postoperative central system infection or aseptic meningitis. Regarding laboratory findings, the blood count underwent a significant early-stage change after surgery but later normalized, showing no correlation with the probe. Simultaneously, no liver or kidney toxic reactions associated with the probe were observed (**Figure S2**).

## Discussion

This study has demonstrated the feasibility of the novel dual-modality ^68^Ga-IRDye800CW-BBN PET/NIR imaging technique in patients with LGGs. Preoperative PET imaging with the same target as the intraoperative optical probe presented a superior predictive value for intraoperative fluorescence than MRI enhancement images. Furthermore, the intraoperative fluorescence showed a higher sensitivity than 5-ALA in guiding precise sampling for patients with LGGs. Additionally, regions exhibiting fluorescence presented a more increased proliferation index; therefore, these regions should be sampled to ensure accurate histopathological diagnosis and grading.

Due to the low sensitivity of intraoperative fluorescence in LGGs, preoperative imaging plays a crucial role in identifying suitable candidates for fluorescence-guided surgery. However, the predictive value of preoperative MRI for intraoperative fluorescence in LGGs has been a subject of controversy 6, 12, 14, 33, 34. To address this, we designed a dual-modality imaging probe for integrating pre- and intraoperative targeted imaging via the same molecular receptor, expecting a higher predictive value for intraoperative fluorescence. Surprisingly, the preoperative ^68^Ga-IRDye800CW-BBN PET uptake presented significantly higher accuracy than contrast-enhanced MRI in predicting intraoperative fluorescence (100.0% *vs.* 88.6%). Our findings suggest that the ^68^Ga-IRDye800CW-BBN PET uptake could be considered a reliable surrogate marker for intraoperative fluorescence in LGGs. If patients show negative PET uptake, the likelihood of intraoperative fluorescence is relatively low, and as a result, the fluorescence-guided resection procedure may not be initiated in such cases.

To date, the potential role of fluorescence-guided surgery in the surgery of LGGs remains incompletely defined. It is widely acknowledged that the currently used fluorescent agents exhibit extremely low sensitivity in LGGs 5, 12, 35. In our series, the introduction of a tumor-targeted fluorescent probe with NIR imaging techniques has expanded the feasibility of fluorescent-guide surgery in LGGs. In comparison to 5-ALA 4, 5, 12, 14, the most commonly utilized optical imaging agent, IRDye800CW-BBN, demonstrated a higher sensitivity of visible fluorescence during surgery (88.6% in WHO grade III gliomas; 54.7% in grade II gliomas). The specific binding between BBN and GRPR on glioma cells facilitates the accumulation of fluorophores within tumor tissue 36, 37. Combining BBN with IRDye800CW introduces NIR imaging technologies, significantly enhancing the spatial resolution of fluorescence-guided surgery. NIR fluorophores, emitting longer wavelengths, enable more sensitive visualization compared to 5-ALA by minimizing the impact of autofluorescence and light scattering while enhancing tissue penetration 15, 24, 38. Consequently, the tumor-specific NIR fluorophore probe emerges as a promising candidate for tumor visualization in the surgery of LGGs.

In the surgery of LGGs, a significant clinical challenge for neurosurgeons lies in identifying tumor tissue, as there is minimal distinction in appearance between tumor and non-tumor tissues under white-light microscopy 7, 39. Fluorescence is an essential guide for tissue sampling during surgery, and precise localization of the fluorescence signal and tumor tissues is vital for accurate tumor sampling 15, 40. In this study, over 90% of samples identified with visual fluorescence were confirmed with positive pathology, higher than the performance of 5-ALA fluorescence-guided sampling in LGGs surgery 7, 41. Therefore, when suspicious tumor lesions exhibit visual fluorescence, surgeons could reasonably conclude that these lesions are tumors and can be sampled and resected. Theoretically, the tumor-specific NIR probe should provide superior tumor visualization compared to 5-ALA in LGGs; however, our study did not directly compare the sensitivity and specificity of fluorescence-guided pathological sampling. Additional randomized controlled trials are necessary to establish more robust evidence.

In the subsequent analysis, we delved into the quantitative fluorescence intensity of samples* ex vivo*, establishing a threshold to differentiate tumor lesions from non-tumor tissues. While this technique provides a semi-quantitative measure of fluorescence intensity rather than absolute fluorophore concentration levels, it significantly bolsters the sensitivity, particularly in WHO grade II samples. This implies there may be an accumulation of IRDye800CW-BBN in specific biopsy samples, but this accumulation might not be sufficient to generate visible fluorescence. We speculate that the inadequate accumulation of fluorophore probes may be attributed to the incomplete disruption of the blood-brain barrier (BBB) in LGGs. As a peptide, the large molecular size of BBN poses challenges in traversing the incomplete BBB disruption 15. Moreover, the full potential advantage of IRDye800CW in intraoperative fluorescence imaging was not realized, as the imaging system employed NIR-I windows rather than NIR-II windows 37, 38. Additionally, subjective methods, such as relying solely on a surgeon's impression in fluorescence analysis, might be prone to bias, particularly in samples with indistinct fluorescence 7, 41. The probe and camera system for intraoperative tumor visualization is still in development, such as small molecular-sized tumor-targeted agents and real-time quantitative fluorescence NIR-II imaging systems, which may further enhance the accuracy of fluorescence-guided sampling in pure LGGs 15, 25, 38.

Another obstacle to pathological sampling for LGGs is the high degree of tumoral heterogeneity. Precisely determining the tumor grade is paramount for tailoring individualized treatment strategies and enhancing patient outcomes 7, 42. The Ki-67/MIB-1 index, associated with proliferation, aids in grading malignancy in gliomas 7, 14. In this investigation, we observed that the Ki-67/MIB-1 index in fluorescence-positive samples was significantly higher than in negative samples, indicating that fluorescence may indicate lesions with the biological potential for malignant transformation. Importantly, we also observed a much shorter time to PFS and OS in patients with intraoperative fluorescence, particularly those with residual fluorescent foci. These findings underscore that intraoperative fluorescence serves as a marker for tumors exhibiting malignant biological behaviors. Biopsies of such areas should be performed specifically, if safely possible, and resected to optimize tissue sampling and improve overall patient management.

This PET/NIR dual-modality probe exhibited promise in enhancing preoperative surgical planning in fluorescence-guided surgery and improving the sensitivity to LGGs visualization during surgery. A subsequent multicenter trial is necessary to obtain most suitable indications of this technique, i.e., which subgroup of LGG patients can benefit in which situation. Stricter designed randomized controlled trail which thoroughly balanced influential factors for survival and bias will be warranted.

## Conclusions

In conclusion, our study successfully demonstrated the potential value of the novel dual-modality PET/NIR imaging probe, ^68^Ga-IRDye800CW-BBN, in LGGs visualization. Preoperative PET assessment offered a useful approach to identifying patients suitable for fluorescence-guided surgery by targeting the same tumor-specific receptor. This tumor-targeted NIR optical probe provided relatively high accuracy in visualizing anaplastic foci of LGGs, thus the intraoperative fluorescence can be a valuable marker for targeted biopsies and, if safely possible, specific resection of such areas.

## Supplementary Material

Supplementary figures.

## Figures and Tables

**Table 1:** Baseline Clinical Characteristics

	No. (%)		
Characteristic	Non-fluorescence (N=14)	Fluorescence (N=25)	*P* Value
Age, mean (SD), yr	39.3 (10.5)	45.8 (10.8)	0.035
Sex			
male	7(50.0)	11 (44.0)	0.750
female	7 (50.0)	14 (56.0)	
Preoperative KPS			
≥90	9 (64.3)	8 (32.0)	0.130
80-90	4 (28.6)	10 (40.0)	
<80	1 (7.1)	7 (28.0)	
Volume, mean (SD), ccm	50.6 (26.3)	87.2 (51.8)	0.019
Location			
non-eloquent	5 (35.7)	5 (20.0)	0.327
near-eloquent	7 (50.0)	11 (44.0)	
eloquent	2 (14.3)	9(36.0)	
Adj. Cytotoxic Therapy			
None	1 (7.1)	2 (8.0)	0.904
Chemo	1 (7.1)	4 (16.0)	
RT	9 (64.3)	13(52.0)	
Chemo+RT	3 (21.4)	6 (24.0)	
Histology			
Astrocytoma, IDH-mutant, CNS 2	7(50.0)	6 (24.0)	0.004
Oligodendrogliomas, IDH-mutant, and 1p19q-codeleted, CNS 2	2 (14.3)	1 (4.0)	
Astrocytoma, NEC, CNS 2	3 (21.4)	1 (4.0)	
Astrocytoma, IDH-mutant, CNS 3	0 (0.0)	4 (16.0)	
Oligodendrogliomas, IDH-mutant, and 1p19q-codeleted, CNS 3	1 (7.1)	12 (48.0)	
Astrocytoma, NEC, CNS 3	1 (7.1)	1 (4.0)	
Molecular markers			
Ki-67/MIB-1, mean (SD), %	8.3 (5.7)	21.0 (12.5)	0.001
IDH status			
wide type	2 (14.3)	4 (16.0)	1.000
mutation	12 (85.7)	21 (84.0)	
1p19q status			
wide type	10 (71.4)	12 (48.0)	0.193
co-deletion	4 (28.6)	13(52.0)	
MGMT meth. Status			
yes	13 (92.9)	22 (88.0)	1.000
no	1 (7.1)	3 (12.0)	

Abbreviation: KPS, Karnofsky performance scale; EOR, extent of resection; Chemo, chemotherapy; RT, radiotherapy
