# Supplementary material for: Lower-grade gliomas surgery guided by GRPR-targeting PET/NIR dual-modality image probe: a prospective and single-arm clinical trial
Source: Theranostics. 2024 Jan 1;14(2):819–29. doi: 10.7150/thno.91554 (PMC10758047; doi:10.7150/thno.91554)
Supplement: Supplementary file 1 — Supplementary figures. [file thnov14p0819s1.pdf]

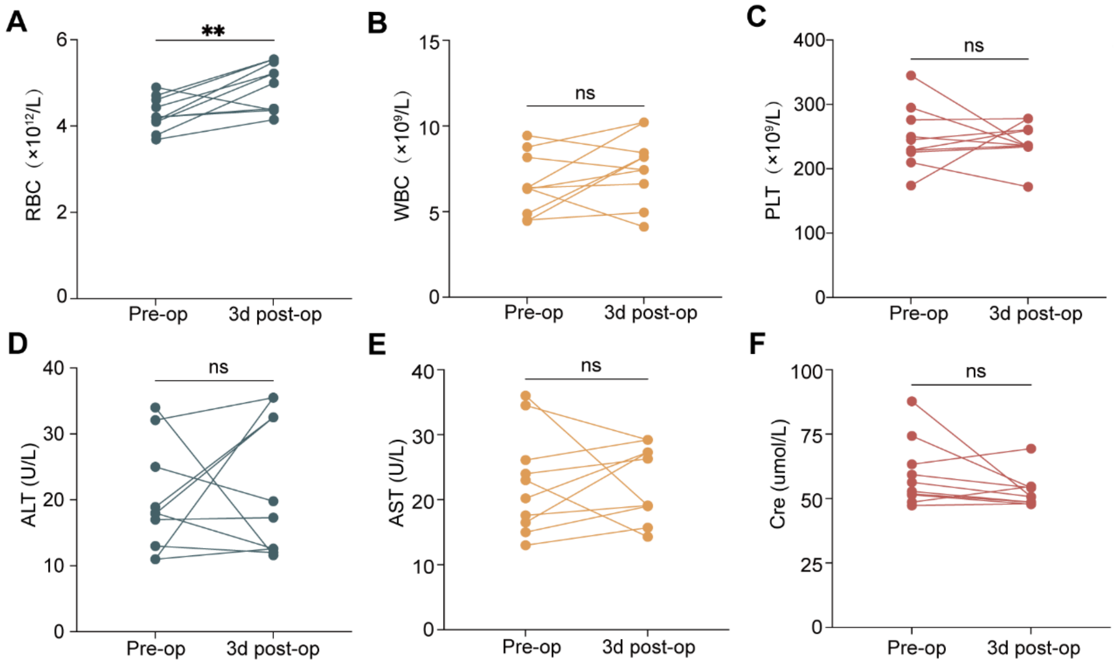

Figure S1. Laboratory tests before and after ^{68}Ga-IRDye800CW-BBN administration (n = 10).

A. Red blood cell count (RBC; reference: 3.8 - 5.1 $\times 10^{12}$/L). B. White blood cell count (WBC; reference: 3.5 - 9.5 $\times 10^{9}$/L). C. Platelet count (PLT; reference: 125 - 350 $\times 10^{9}$/L). D. Glutamic-pyruvic transaminase (ALT; reference: 0 - 41 U/L). E. Glutamic-oxaloacetic transaminase (AST; reference: 0 - 42 U/L). F. Creatinine (Cre; reference: 31.7 - 93.3 μmol/L). **, $P < .05$; ns, $P > .05$ not significant.

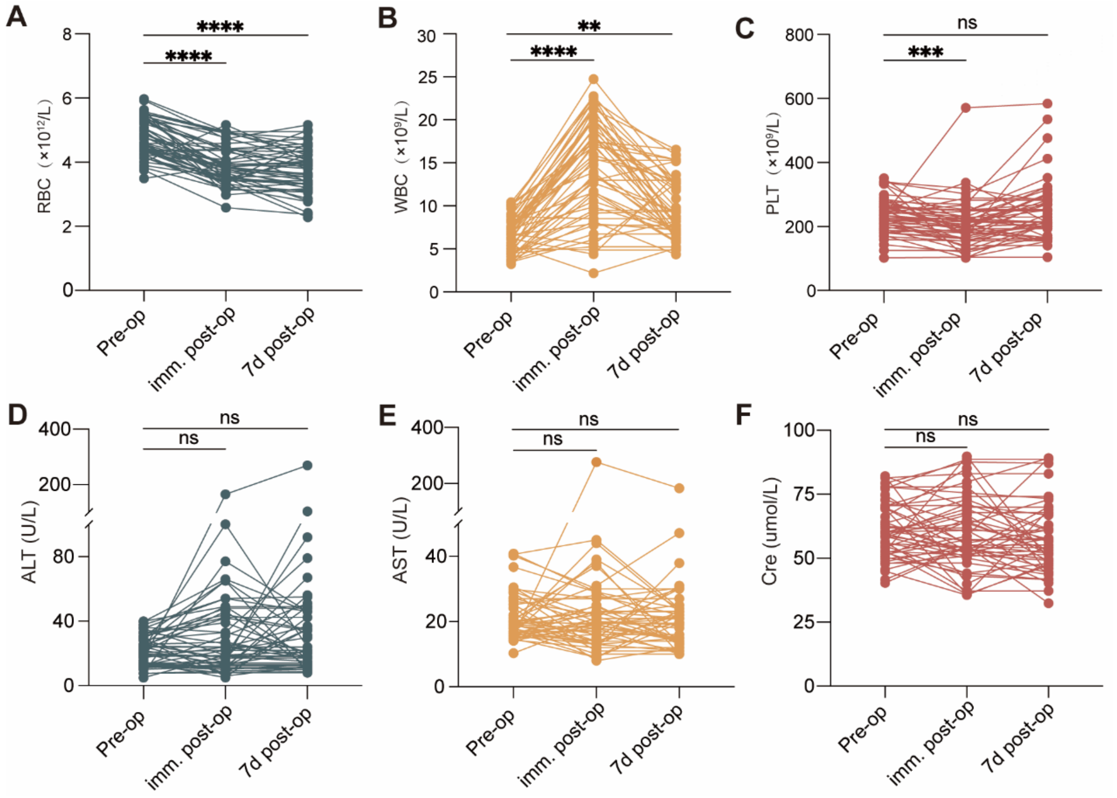

Figure S2. Laboratory tests before and after IRDye800CW-BBN administration (n = 52).

A. Red blood cell count (RBC; reference: 3.8 - 5.1 $\times 10^{12}$/L). B. White blood cell count (WBC; reference: 3.5 - 9.5 $\times 10^{9}$/L). C. Platelet count (PLT; reference: 125 - 350 $\times 10^{9}$/L). D. Glutamic-pyruvic transaminase (ALT; reference: 0 - 41 U/L). E. Glutamic-oxaloacetic transaminase (AST; reference: 0 - 42 U/L). F. Creatinine (Cre; reference: 31.7 - 93.3 μmol/L). **, $P < .05$; ***, $P < .01$, ****, $P < .001$; ns, $P > .05$ not significant.
